# Editorial: Opportunities and challenges of interprofessional collaboration and education

**DOI:** 10.3389/fmed.2024.1392690

**Published:** 2024-03-21

**Authors:** Jill E. Thistlethwaite, Peter Musaeus, Martina Müller

**Affiliations:** ^1^Faculty of Health, University of Technology Sydney, Sydney, NSW, Australia; ^2^School of Nursing and Midwifery, Western Sydney University, Penrith, NSW, Australia; ^3^Centre for Educational Development, Aarhus University, Aarhus, Denmark; ^4^University of Regensburg, Regensburg, Bavaria, Germany

**Keywords:** interprofessional collaboration, interprofessional education, interprofessional collaborative practice, interprofessional collaborative practice competencies, interprofessional learning

In the contemporary era, health and social care are delivered by different professionals who engage with patients, clients, families, and communities. These encounters require interprofessional collaborative practice (IPCP) defined as “the process of developing and maintaining effective interprofessional working relationships with learners, practitioners, patients/clients/families and communities to enable optimal health outcomes” (1). IPCP is thus quality care that does not happen automatically by involving different professionals but requires attention to many factors including location, context, methods of communication, level of understanding of team roles, team tasks, professional backgrounds, scopes of practice, and patient experiences. Due to the intricate web of social interactions required, IPCP can be extremely challenging.

Collaboration encompasses teamwork and in addition other models of interprofessional working that occur in contemporary health care systems. It is a complex process that is not confined to person to person social interaction such as, for example, between nurse and patient, but rather interactions amongst health and social care organizations, teams, and professionals working to ensure a patient's trajectory in the healthcare system is as efficient and humane as possible. Thus, IPCP is relevant not only in individual social interactions but also broadly within societies' health and social care systems. IPCP can be conceived in terms of regulated agents as: “a process in which autonomous or semi-autonomous actors interact through formal and informal negotiation, jointly creating rules and structures governing their relationships and ways to act or decide on the issues that brought them together; it is a process involving shared norms and mutually beneficial interactions” (2). Six elements influencing interprofessional working have been identified: shared team identity; clear roles/goals; interdependence; integration; shared responsibility; and team tasks (3). These occur in various degrees from co-located teamwork with a few members to much wider networks with lesser levels of interdependence, integration and shared responsibility as the number of people involved increases.

Preparing professionals for collaboration requires specific training, including learning from self and others. As the title of a World Health Organization publication from 1988 succinctly states this involves “learning together to work together” (4). The Centre for the Advancement of Interprofessional Education's (CAIPE) definition of interprofessional education (IPE) is “occasions when two or more professions learn with, from and about each other, to improve collaboration, and the quality of care” (5). The definition stresses the interactive nature of interprofessional learning (IPL) that may be formal, informal or serendipitous. IPL should involve more than simple observation of professionals at work, for example a medical student sitting in with a nurse practitioner, but rather participation, simulation and, if feasible, authentic real-life placements where learners become part of the team. There is a burgeoning literature on the contextual nature of IPC and factors leading to successful IPE.

The field of interprofessional education and collaborative practice (IPECP) research and evaluation is expanding. The global network, InterprofessionalResearch. Global (IPR.Global), has proposed three areas for IPECP research that include building the science and scholarship of IPECP, addressing the complexity of interprofessional endeavors through innovative approaches, and developing evidence of impact along the continuum from IPE to service delivery (6). These areas encompass health professional education, practice, and the connection between them.

In this Frontiers in Medicine, Healthcare Professions Education, Research Topic on *Opportunities and challenges of interprofessional collaboration and education*, the collection of 13 papers covers a range of topics related to IPECP but primarily focuses on IPE. Seven are of German authorship, reflecting in part the growth of IPE in Germany and indeed the other German-speaking countries of Austria and Switzerland. These three countries have formed a regional network, IP-Health (Society for Interprofessional Health and Social Care), which is a member of Interprofessional.Global (www.interprofessional.global).

At the forefront of authentic practice-based IPE for health professional students are the interprofessional training wards (IPTW), which are functioning inpatient wards staffed by students working collaboratively under supervision. The first documented IPTW was opened in Sweden in 1996 (7). The first German IPTWs were implemented in 2017 (8). Evaluation of such wards contributes to our knowledge of the impact of IPE on student learning and factors contributing to such learning, and three papers from Germany on IPTWs are included in this Research Topic. Mitzkat et al. report on the development of individual competencies and team performance of medical and nursing students on placement in the Heidelberg IPTW. Straub et al. studied an IPTW in pediatrics in Freiburg and its effectiveness in training nursing and medical students. A questionnaire was developed to evaluate students' learning experiences and program structure. In their study on patient perspectives within an IPTW in internal medicine in Regensburg, Schlosser-Hupf et al. found that the clinical impact of these educational structures was significant, with 96.7% of patients appreciating the ward rounds' atmosphere and conduct, and 98.3% satisfied with treatment discussions and information during their hospital stay.

In addition, Albrecht et al. discuss that health professionals such as physicians and nurses contribute significantly to the transformation process toward a healthy, sustainable and climate-sensitive society. The results of their survey suggest that the current state of climate-specific health literacy differs between different groups of health professionals. They conclude that there is a need to improve health professionals' levels of climate-specific health literacy and that IPCP and IPTWs play an important role in increasing awareness and knowledge regarding planetary health.

Other IPL activities included in this Research Topic focus on learning through adaptation and simulation (De Wever et al.) and during student placements on international electives (Nawagi et al.). Specific areas for IPL covered in the collection include care of patients with dementia (Dressel et al.), and point-of-care ultrasound (POTUS) for post-licensure emergency department team-based health professionals (Witte et al.). These papers highlight the need for interactive learning and planning by an interprofessional team.

IPL should be focussed on helping students to meet defined interprofessional competencies or learning outcomes. There are several interprofessional competency frameworks that can assist in such definition, two of the most cited being those of the Canadian Interprofessional Health Collaborative of 2010 (1), currently being updated, and the Interprofessional Education Collaborative Expert Panel, updated in 2023 (9). However, in recognition of diverse populations and health systems globally, some jurisdictions devise their own lists to meet the needs of their local communities. Andersen et al. present their synthesis of national expert opinion on interprofessional competency indicators for health professional students in New Zealand particularly in public health promotion.

Another important factor in IPE is faculty development: training of academic staff for interprofessional facilitation. Schlicker et al. consider the challenges of introducing IPE in Germany when educators are insufficiently prepared and advocate for specific training that includes interprofessional learning for the educators themselves, with two or more professions learning together to develop IPE competencies.

Given that healthcare communication is complex, interprofessional communication is perhaps even more complicated. There is a danger of poor health communication (between health professionals or between health professional and patient). Therefore there is a need for research on what is good interprofessional communication and the mechanisms explaining why some modes of communication are more efficient than others. This is addressed in two scoping reviews. First, in a scoping review on distributed team processes in healthcare services, Eid et al. identify the need for improved communication and coordination, especially in geographically dispersed settings. Also, the study emphasizes the need for more (longitudinal as well as intervention-control) research, particularly from low- and middle-income countries. In the study by Abu-Rish Blakeney et al. it was found that poor communication in healthcare leads to inefficiencies, errors, and conflicts. In this study a model is proposed on how to involve multiple healthcare professions, patients, and families in collaborative care planning. Research and evaluation of IPE benefits from interdisciplinary as well as interprofessional input: exploring the utilization of concepts and ways of working from disciplines not traditionally associated with health care. Ferreira et al. advocate for the application of systems engineering (SE) to help manage and sustain the complexity of IPE and its aim of improving patient care through interprofessional collaboration.

The World Health Organization in 2010 concluded that one of the most promising solutions in view of the megatrends in healthcare can be found in interprofessional collaboration (10). We extend our gratitude to all researchers across various disciplines who have contributed their work to our Research Topic of interprofessional collaboration and education. We hope our readers find valuable insights and benefits for their research, teaching, and clinical practices, further enriching this important field.

## Author contributions

JT: Writing—review & editing, Writing—original draft. PM: Writing—review & editing, Writing—original draft. MM: Writing—original draft, Writing—review & editing.
